# Dairy Cows Naturally Infected with Bovine Leukemia Virus Exhibit Abnormal B- and T-Cell Phenotypes after Primary and Secondary Exposures to Keyhole Limpet Hemocyanin

**DOI:** 10.3389/fvets.2017.00112

**Published:** 2017-07-14

**Authors:** Meredith C. Frie, Kelly R. B. Sporer, Oscar J. Benitez, Joseph C. Wallace, Casey J. Droscha, Paul C. Bartlett, Paul M. Coussens

**Affiliations:** ^1^Cell and Molecular Biology Program, Michigan State University, East Lansing, MI, United States; ^2^Department of Animal Science, Michigan State University, East Lansing, MI, United States; ^3^Comparative Medicine and Integrative Biology Program, Michigan State University, East Lansing, MI, United States; ^4^NorthStar Cooperative, East Lansing, MI, United States; ^5^Department of Large Animal Clinical Sciences, Michigan State University, East Lansing, MI, United States

**Keywords:** bovine leukemia virus, memory, CD5^+^ B cells, gamma delta T cells, CD45R0, IFNγ, IL4, antibody

## Abstract

Bovine leukemia virus (BLV) is a retrovirus that is highly prevalent in US dairy herds: over 83% are BLV infected and the within-herd infection rate can be almost 50% on average. While BLV is known to cause lymphosarcomas, only 5% or fewer infected cattle will develop lymphoma; this low prevalence of cancer has historically not been a concern to dairy producers. However, more recent research has found that BLV^+^ cows without lymphoma produce less milk and have shorter lifespans than uninfected herdmates. It has been hypothesized that BLV infection interferes with normal immune function in infected cattle, and this could lead to reduced dairy production. To assess how naturally infected BLV^+^ cows responded to a primary and secondary immune challenge, 10 BLV^+^ and 10 BLV^−^ cows were injected subcutaneously with keyhole limpet hemocyanin (KLH) and dimethyldioctadecylammonium bromide. B- and T-cell responses were characterized over the following 28 days. A total of 56 days after primary KLH exposure, cows were re-injected with KLH and B- and T-cell responses were characterized again over the following 28 days. BLV^+^ cows produced less KLH-specific IgM after primary immune stimulation; demonstrated fewer CD45R0^+^ B cells, altered proportions of CD5^+^ B cells, altered expression of CD5 on CD5^+^ B cells, and reduced MHCII surface expression on B cells *ex vivo*; exhibited reduced B-cell activation *in vitro*; and displayed an increase in BLV proviral load after KLH exposure. In addition, BLV^+^ cows had a reduced CD45R0^+^γδ^+^ T-cell population in the periphery and demonstrated a greater prevalence of IL4-producing T cells *in vitro*. All together, our results demonstrate that both B- and T-cell immunities are disrupted in BLV^+^ cows and that antigen-specific deficiencies can be detected in BLV^+^ cows even after a primary immune exposure.

## Introduction

Bovine leukemia virus (BLV) is a δ-retrovirus (1) that infects over 83% of dairy herds in the United States; as many as 40% of all US dairy cattle are infected (2). BLV is the causative agent of enzootic bovine leukosis (EBL), where BLV^+^ cattle develop malignant lymphoma or leukemia (3). Although lymphoma is deadly and results in carcass condemnation at slaughter (2), it is estimated that fewer than 10% of infected cattle will eventually develop EBL (3). Unfortunately, recent research suggests that BLV infection has more negative impacts on herd health than previously appreciated.

Bovine leukemia virus infection reduces both milk production (2) and longevity (4) in infected dairy cows. While it is unclear how BLV interferes with milk production and lifespan, one hypothesis is that BLV causes immune dysregulation, which could put BLV^+^ cattle at an increased risk for other infections. BLV most commonly infects B cells (5) and can cause persistent lymphocytosis (PL), a chronic, benign, polyclonal expansion of the B-cell compartment that occurs in 30% of infected cattle (3). *In vitro* experiments have demonstrated abnormalities in both innate and adaptive immune cells isolated from BLV^+^ cattle (6). In addition, a few studies have found positive correlations between BLV and other infectious diseases (7, 8) and a reduction in vaccine immunity in BLV^+^ cattle (9–11). However, when investigating immunity in naturally infected BLV^+^ cattle, many studies were unable to control for how much antigen exposure occurred before or after BLV infection.

The current study was designed to address that specific problem. BLV^+^ and BLV^−^ cows were exposed to an immunostimulatory antigen, keyhole limpet hemocyanin (KLH), to mimic a primary immune response. At 56 days after primary exposure, cows were re-exposed to KLH to mimic a secondary memory immune exposure. To characterize both primary and secondary adaptive immune responses, B- and T-cell responses were tracked using ELISAs to measure antibody production against KLH, flow cytometry to measure the dynamics of freshly isolated B and T cell subsets, and cell culture to investigate B- and T-cell responses to KLH and mitogenic stimulation *in vitro*. Specifically, CD5 and CD45R0 expressions on *ex vivo* B cells and CD45R0 expression on *ex vivo* CD4^+^, CD8^+^, and γδ^+^ T cells were characterized. BLV and CD25 expressions were characterized in B cells, and IFNγ and IL4 productions were characterized in T cells after *in vitro* stimulation. Abnormalities in both B- and T-cell subsets were detected in BLV^+^ cattle during both primary and secondary immune responses, providing further support that BLV infection causes immune dysregulation.

## Materials and Methods

### Animals and KLH Inoculation

10 BLV^−^ and 10 BLV^+^ lactating Holstein dairy cows were enrolled in the current study (Table 1). BLV^+^ cows (as determined by the producer’s BLV milk ELISA results) were not confirmed to have PL but were selected for elevated total leukocyte counts (as determined using a Beckman Coulter counter) and an elevated proportion of circulating B cells [as determined by immunostaining for surface IgM (SIgM) on freshly isolated PBMCs] 1 week prior to the study’s initiation. BLV^+^ cows had a high proviral load (PVL) on d0 (12). BLV^−^ cows were then age and lactation matched to the 10 selected BLV^+^ cows. Both BLV^−^ and BLV^+^ cows were also re-screened for BLV infection using a commercial serum ELISA (NorthStar Cooperative) 1 week prior to the study start. BLV serum ELISAs and endpoint PCR (on DNA extracted from whole blood) to detect BLV provirus were also used on samples collected on the first and last days of the study to confirm BLV status. One BLV^−^ cow seroconverted in between enrollment diagnostics and the start of the study; this cow and her matched BLV^+^ cow were excluded from the final data analysis.

**Table 1:** Cow enrollment characteristics.

	BLV^−^ cows	BLV^+^ cows
Age	4 years 11 months (3 years 7 months–6 years 11 months)	4 years11 months (3 years 7 months–7 years 5 months)
Days in milk	203.5 (140–350)	197.7 (127–293)
Lactation number	3.3 (2–5)	3.2 (2–5)
Reproduction status	All pregnant	All pregnant
%B cell	37.89 (29.65–48.59)	55.81 (40.96–72.86)
Proviral load per 100,000 cells	N/A	95,879 (62,247–123,429)

*Data are presented from d0. Data are represented as the mean and (range)*.

Upon study initiation on day 0 (d0), all cows received the primary KLH inoculation consisting of 1.5 mL KLH cocktail injected subcutaneously into the left side of the neck. The KLH cocktail was composed of 200 µg KLH (Sigma) in 0.75 mL 1× phosphate-buffered saline (PBS) containing 5% bovine serum albumin and 0.75 mL 20 mg/mL adjuvant dimethyldioctadecylammonium bromide (DDA) (Sigma) in 1× PBS (13). On d56, cows received the secondary KLH inoculation with 1.5 mL KLH cocktail subcutaneously injected into the right side of the neck. All protocols were reviewed and approved by the Michigan State University Institutional Animal Use and Care Committee (AUF# 04/15-061-00).

### Whole Blood, Plasma, and PBMC Isolation

Whole blood was collected by coccygeal venipuncture on d0 before primary inoculation and on days 7, 14, 18, 21, 28, 54, and 56 after primary inoculation; after blood collection on d56, the secondary KLH inoculation was administered and blood was collected on days 60, 67, 70, 77, and 84 after primary inoculation (days 4, 11, 14, 21, and 28 after secondary inoculation). Blood for PVL quantification was collected in Vacutainer blood collection tubes containing the anticoagulant EDTA (Becton Dickinson); 1 mL aliquots of whole blood were stored at −80°C. Blood for antibody quantification and PBMC immunostaining was collected in Vacutainer blood collection tubes containing the anticoagulant ACD (Becton Dickinson). Plasma and PBMCs were isolated as previously described (10). Briefly, aliquots of plasma with 0.1% sodium azide were stored at −80°C and PBMCs were isolated using Percoll density centrifugation.

### BLV PVL Quantification

DNA was extracted from whole blood using the DNeasy Blood and Tissue kit (Qiagen) using a modified protocol. Briefly, 200 µL of whole blood, 40 µL of proteinase K, 218 µL of buffer AL and 218 µL of 100% ethanol were used instead of the recommended kit volumes. Extracted DNA was quantified using a Nanodrop-1000, and A260/280 ratios were used to assess sample purity. DNA was diluted to 30 ng/µL in elution buffer for PVL quantification. BLV PVL was determined using the Coordination of Common Motifs-qPCR to amplify the long terminal repeat (LTR) of the BLV provirus. To normalize genomic DNA input, the single-copy Bola-DRA gene was also amplified (14). In brief, 30 ng of genomic DNA were assayed using TaqMan Gene Expression Master Mix (Applied Biosystems, CA, USA) on the 7500 FAST Real-time PCR System (Applied Biosystems, CA, USA). BLV copy number and BoLA-DRA copy number were calculated using 10–1 × 10^5^ copies of the standard plasmid, which contained a copy of BLV-LTR and BoLA-DRA. Each value was calculated using the algorithm suggested by the manufacturer. PVL was the ratio of BLV copy number to BoLA-DRA copy number multiplied by 100,000 (15). PVL was expressed as BLV copy number/100,000 cells.

### Anti-KLH Antibody Quantification

Anti-KLH antibodies were quantified from plasma collected on days 0, 7, 14, 21, 28, 56, 60, 67, 70, 77, and 84 after primary inoculation. Flat-bottomed 96-well ELISA plates (Thermo Fisher Scientific) were incubated with 100 µL of 1 µg/mL KLH in 50 mM carbonate/bicarbonate buffer overnight at 4°C. Plates were washed 3× with wash buffer (0.05% Tween-20 in 1× PBS) and blocked with 2% heat-inactivated horse serum in wash buffer for 1 h at 37°C. Plates were then washed 5×, and 100 µL of plasma (diluted 1:50 in blocking buffer) was added to wells and incubated for 1 h at room temperature. Plates were washed 5× and then incubated for 1 h at room temperature with 100 µL anti-bovine IgM, IgG1, or IgG2 conjugated to horseradish peroxidase (Thermo Fisher Scientific) diluted 1:10,000 in blocking buffer as previously described (10). Plates were washed 5× and incubated for 15 min at room temperature in the dark with 100 µL TMB substrate (Sigma). A total of 100 µL stop solution (2 M H_2_SO_4_) was added, and the optical density was measured at 450 nm using a SpectraMax M5 microplate reader. All samples were run in duplicate, and each plate included blank and naive (not exposed to KLH) controls.

### Immunostaining of Freshly Isolated PBMCs

Immunostaining of freshly isolated PBMCs was performed as previously described (10) on days 0, 7, 14, 21, 28, 56, 60, 67, 70, and 77 after primary inoculation. Briefly, 5 × 10^5^ PBMCs were fixed in 4% paraformaldehyde and then labeled via indirect immunofluorescence first with mouse anti-bovine primary antibodies and second with goat anti-mouse secondary antibodies. The freshly isolated B-cell population was characterized using the stain labeled “fresh B” in Table 2, and the freshly isolated T-cell population was characterized using the stain labeled “fresh T” in Table 2.

**Table 2:** Antibodies used for immunostaining experiments.

Stain	1° MAb target	Target phenotype	Clone	2° Ab target
Fresh B	CD45R0	Effector/memory lymphocytes	ILA116A	IgG3 AF88
	MHCII	Antigen presentation	TH16A	IgG2a PE
	Surface IgM (SIgM)	B cells	PIG45A	IgG2b PE-Cy7
	CD5	CD5^+^ B cells	CACT105A	IgG1 AF647
Fresh T	CD45R0	Effector/memory lymphocytes	ILA116A	IgG3 AF88
	CD4	Helper T cells	IL11A	IgG2a PE
	CD8	Cytotoxic T cells	BAQ111A	IgM PE-Cy7
	γδ TcR	Gamma delta T cells	GB21A	IgG2b AF647
IFNγ	γδ TcR	Gamma delta T cells	GB21A	IgG2b AF88
	CD4	Helper T cells	IL11A	IgG2a PE
	CD8	Cytotoxic T cells	BAQ111A	IgM PE-Cy7
	IFNγ^a^	Th1 cytokine	CC302	IgG1 AF647^a^
IL4	γδ TcR	Gamma delta T cells	GB21A	IgG2b AF88
	CD4	Helper T cells	CACT138A	IgG1 PE
	CD8	Cytotoxic T cells	BAQ111A	IgM PE-Cy7
	IL4^a^	Th2 cytokine	CC303	IgG2a AF647^a^
CD45R0	CD45R0	Effector/memory lymphocytes	ILA116A	IgG3 AF88
	MHCII	Antigen presentation	TH16A	IgG2a PE
	SIgM	B cells	PIG45A	IgG2b PE-Cy7
	Bovine leukemia virus (BLV) gp51^a^	BLV-expressing B cells		IgG1 AF647^a^
CD25	CD25	IL2 receptor, α chain	LCTB2A	IgG3 AF88
	MHCII	Antigen presentation	TH16A	IgG2a PE
	SIgM	B cells	PIG45A	IgG2b PE-Cy7
	BLV gp51^a^	BLV-expressing B cells		IgG1 AF647^a^

*All primary MAbs were purchased from Washington State University Monoclonal Antibody Center except IFNγ and IL4 (Bio-Rad) and BLV gp51 (VMRD). All secondary antibodies were purchased from Thermo Fisher Scientific except IgM PE-Cy7 (eBioscience) and IgG2b PE-Cy7 (Abcam)*.

*^a^Antibodies used for intracellular labeling*.

### *In Vitro* Stimulation of PBMCs

To investigate T-cell activation, 2 × 10^6^ PBMCs were cultured at 38°C and 5% CO_2_ in 1 mL Roswell Park Memorial Institute (RPMI) complete media (RPMI plus 10% fetal bovine serum, 1% penicillin/streptomycin, and 1% fungizone, pH 7.4) in 24-well culture plates (Corning). PBMCs were either cultured in medium alone (NIL) for 18 h, with 200 µg/mL KLH for 18 h, or with 20 µg/mL positive control concanavalin A (CONA) for the final 6 h. All samples were treated with 20 ng/mL brefeldin A at 12 h to prevent cytokine secretion. T-cell activation was measured on days 7, 14, 56, 67, and 77.

To investigate B-cell activation, 5 × 10^6^ PBMCs were cultured at 38°C and 5% CO_2_ in 3 mL RPMI complete media in 12-well culture plates (Corning) with medium alone (NIL), 200 µg/mL KLH, or with positive control 20 ng/mL phorbol 12-myristate 13-acetate and 400 ng/mL ionomycin (P/I) for 18 h. B-cell activation was measured on days 18, 54, and 70.

### Immunostaining of Cultured PBMCs

PBMCs were labeled with four-color stains to investigate IFNγ production by T cells (“IFNγ” in Table 2), IL4 production by T cells (“IL4” in Table 2), and BLV expression in B cells (“CD45R0” and “CD25” in Table 2). Immunostaining of cultured PBMCs was performed as previously described (10). Briefly, indirect immunostaining for surface receptors on live PBMCs was done as described for freshly isolated PBMCs. PBMCs were then fixed and permeabilized using a fixation/permeabilization kit according to manufacturer’s instructions (eBioscience), and PBMCs were labeled with a primary mouse anti-bovine or anti-BLV monoclonal antibody and subsequently labeled with a goat anti-mouse secondary antibody. Labeled PBMCs were stored at 4°C overnight until flow cytometry analysis.

### Immunostaining Analysis

Data were acquired using an Accuri C6 flow cytometer equipped with CSampler (Becton Dickinson). Data were compensated using the Accuri C6 software (Becton Dickinson) and then exported for analysis using FCS Express 4 (*De Novo* Software). PBMCs were initially selected using forward and side scatter gating to exclude debris, and cells were identified gating on lineage-specific markers (CD4, CD8, γδ, or SIgM). Expression of IFNγ, IL4, CD45R0, MHCII, CD5, and CD25 were then subsequently characterized within specific cell types, and marker expression was expressed as either the mean relative percent or the mean fluorescence intensity (MFI). Unless noted, all referenced B cells are SIgM^+^MHCII^+^. CD5 labeling on fresh PBMCs exhibited three populations; the center population was denoted “CD5^dim^” and the far right population was denoted “CD5^bright^” (Figure S1 in Supplementary Material).

### Statistics

Statistical analysis was performed using SAS 9.4 (SAS Institute). Antibody and fresh PBMC data were analyzed using repeated measures ANOVA with BLV and time as fixed effects and *post hoc* Bonferroni correction for pairwise comparisons. Cultured PBMC data were analyzed using repeated measures ANOVA with BLV and stimulant as fixed effects and *post hoc* Bonferroni correction for pairwise comparisons. A Tukey test was used to analyze the difference in MHCII or CD25 MFIs between different B-cell subsets. Outliers were detected using Grubbs test; both outliers and their matched cow were eliminated from analysis. Significance was determined as *p* < 0.05.

## Results

### BLV^+^ Cows Exhibit Reduced IgM Production *In Vivo*

**Figure 1:**
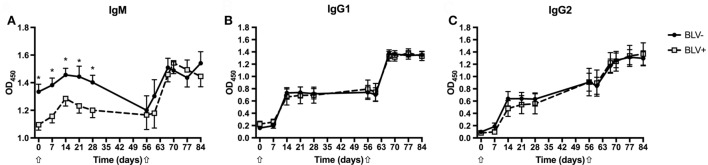
Anti-keyhole limpet hemocyanin (KLH) antibodies produced by BLV^+^ and BLV^−^ cows. Anti-KLH antibodies from BLV^+^ and BLV^−^ cows were relatively quantified. **(A)** IgM production; **(B)** IgG1 production; and **(C)** IgG2 production. *p < 0.05. *n* = 7–9/group. Arrows denote KLH inoculations. Data represent the mean ± SEM.

To characterize the strength of the humoral immune response in BLV^+^ cows, the relative quantities of KLH-specific IgM, IgG1, and IgG2 were measured. As expected, both BLV^+^ and BLV^−^ cows produced KLH-specific IgM, IgG1, and IgG2 after primary and secondary KLH inoculations (*p* < 0.001 for all isotypes). Interestingly, BLV^+^ cows had less IgM (*p* = 0.0045) after primary KLH inoculation at all time points, including d0. However, BLV^+^ cows made IgM at levels equal to BLV^−^ cows after the secondary KLH inoculation (*p* = 0.7742) (Figure 1A). In contrast to IgM production, BLV^+^ and BLV^−^ cows produced equivalent levels of IgG1 (*p* = 0.6715) (Figure 1B) and IgG2 (*p* = 0.9437) (Figure 1C) after both primary and secondary KLH inoculations. These data support recent evidence that BLV infection interferes with IgM antibody production after both a primary immune challenge and a common vaccine booster injection (10).

### BLV^+^ Cows Demonstrate Abnormal Circulating B-Cell Populations *In Vivo*

**Figure 2:**
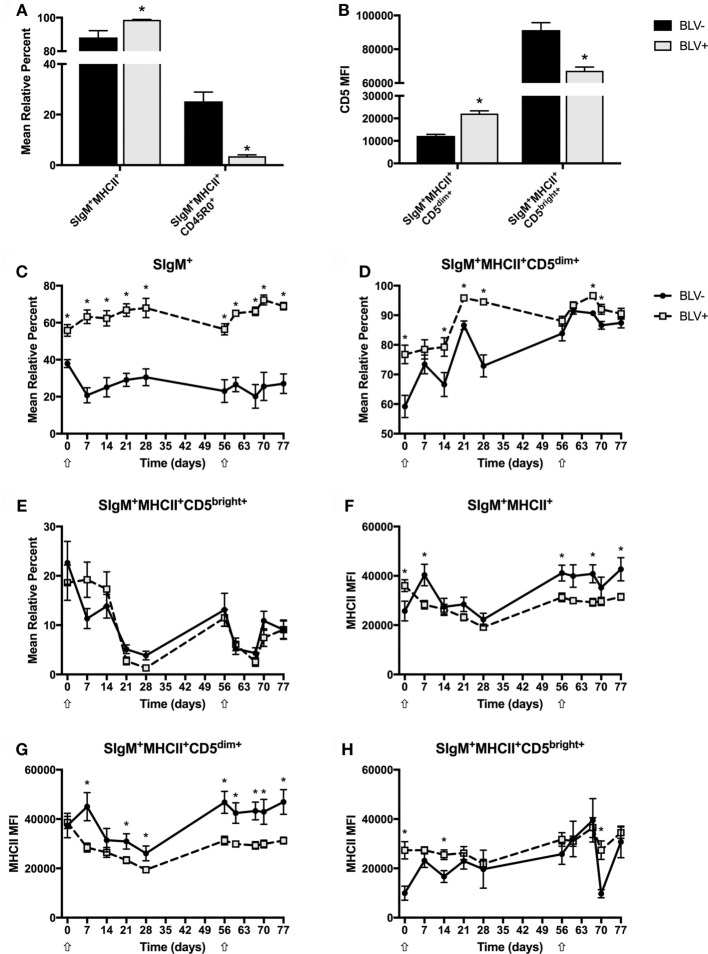
Circulating B-cell populations in BLV^+^ and BLV^−^ cows. Freshly isolated PBMCs from BLV^+^ and BLV^−^ cows were immunostained to characterize the circulating B-cell population. **(A)** Mean relative percentages of B-cell populations on d0. **(B)** Mean fluorescence intensities (MFIs) of B-cell populations on d0. **(C)** Mean relative percent of SIgM^+^ PBMCs. **(D)** Mean relative percent of CD5^dim+^SIgM^+^MHCII^+^ B cells. **(E)** Mean relative percent of CD5^bright+^SIgM^+^MHCII^+^ B cells. **(F)** MHCII MFI on SIgM^+^MHCII^+^ B cells. **(G)** MHCII MFI on SIgM^+^MHCII^+^CD5^dim+^ B cells. **(H)** MHCII MFI on SIgM^+^MHCII^+^CD5^bright+^ B cells. *p < 0.05. *n* = 6–9/group. Arrows denote keyhole limpet hemocyanin inoculations. Data represent the mean ± SEM.

To investigate how circulating B-cell populations changed in response to immune stimulation, freshly isolated PBMCs were immunostained to examine both CD45R0 and CD5 B-cell populations in BLV^+^ and BLV^−^ cows. Unless noted in the text, all B cells are SIgM^+^MHCII^+^. Surprisingly, the SIgM^+^ B-cell population from BLV^+^ cows did steadily increase over time (*p* < 0.05) after both primary and secondary KLH inoculations; however, this trend was not observed in BLV^−^ cows. As expected, BLV^+^ cows had significantly more circulating B cells than uninfected, age-matched cows (*p* < 0.0001) (Figure 2C). Some B-cell populations did not shift in response to KLH inoculation: in both BLV^+^ and BLV^−^ cows, the mean relative percent of MHCII^+^SIgM^+^ B cells and CD45R0^+^ B cells, as well as the CD5 MFI on B cells, all remained constant. However, these populations were altered in BLV^+^ cows. While BLV^+^ cows exhibited a higher proportion of MHCII^+^SIgM^+^ B cells (*p* = 0.0084), they also demonstrated a large reduction in CD45R0^+^ B cells compared to uninfected, age-matched controls (*p* < 0.0001) (Figure 2A). When analyzing CD5 expression on B cells, two distinct positive populations were observed, which we denoted CD5^dim^ and CD5^bright^ (Figure S1 in Supplementary Material). Interestingly, B cells from BLV^+^ cows had higher CD5 expression on CD5^dim+^ B cells (*p* = 0.0002) but lower CD5 expression on CD5^bright+^ B cells (*p* = 0.0017) (Figure 2B).

Although the surface expression of CD5 did not change over time, both CD5^dim+^ and CD5^bright+^ B-cell populations responded to KLH inoculation (*p* < 0.05); while the CD5^dim+^ B-cell population steadily increased after inoculation, the CD5^bright+^ B-cell population sharply declined. In addition, BLV^+^ cows displayed a lower CD5^dim+^ B-cell population (*p* = 0.0043) (Figure 2D), but the CD5^bright+^ B-cell population was equal between BLV^+^ and BLV^−^ cows (*p* = 0.9611) (Figure 2E).

We were also interested in the effect of BLV infection on MHCII surface expression. Surprisingly, MHCII surface expression actually fluctuated in response to KLH exposure on all measured B-cell types (*p* < 0.05). BLV^+^ cows presented lower MHCII surface expression on CD5^dim+^ B cells (*p* = 0.0168) (Figure 2G) and a trending lower MHCII surface expression on all B cells (*p* = 0.0722) (Figure 2F). In contrast, BLV^+^ and BLV^−^ cows exhibited equivalent MHCII surface expression on CD45R0^+^ (*p* = 0.7919) (data not shown) and CD5^bright+^ B cells (*p* = 0.5662) (Figure 2H).

### B Cells from BLV^+^ Cows Develop Atypical Phenotypes after *In Vitro* Stimulation

To determine if KLH stimulation *in vitro* induced BLV expression in infected B cells, PBMCs were cultured in the presence of KLH or P/I-positive control stimulation and BLV expression (by immunostaining for viral protein BLV gp51) and B-cell activation (by immunostaining for CD25) were characterized. While B-cell culture was done on d18, 54, and 70, there was no difference in results from different time points. Thus, results presented are from d70.

**Figure 3:**
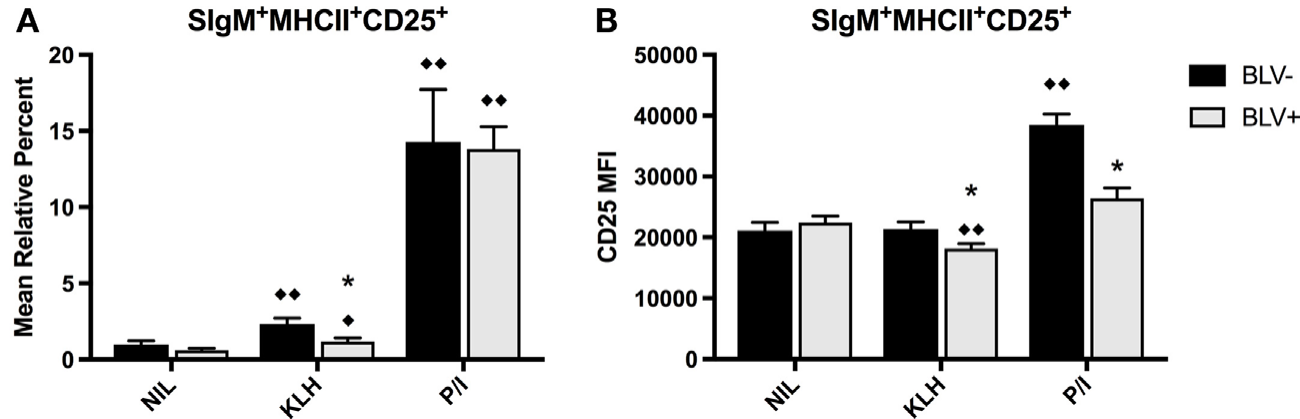
B-cell activation in BLV^+^ and BLV^−^ cows after *in vitro* stimulation. PBMCs from BLV^+^ and BLV^−^ cows were cultured in the presence of no (NIL), keyhole limpet hemocyanin (KLH), or positive control (P/I), stimulation and activation was measured by CD25 expression. **(A)** Mean relative percent of CD25^+^SIgM^+^MHCII^+^ B cells. **(B)** CD25 mean fluorescence intensity on SIgM^+^MHCII^+^CD25^+^ B cells. *p < 0.05 compared to BLV^−^, ^♦♦^*p* < 0.05 compared to nil, ^♦^*p* < 0.1 compared to nil. *n* = 8–9/group. Data represent the mean ± SEM.

There was no overall difference between BLV^+^ and BLV^−^ cows when comparing the mean relative percent of CD25^+^ B cells (*p* = 0.5849), and both KLH and P/I stimulation increased the proportion of CD25^+^ B cells. However, B cells from BLV^+^ cows exhibited only a trending (*p* = 0.053) rise in CD25^+^ B cells after KLH stimulation; in fact, the proportion of CD25^+^ B cells in KLH-stimulated cultures from BLV^+^ cows was significantly lower than KLH-stimulated cultures from BLV^−^ cows (Figure 3A).

In contrast to the mean relative percent of activated B cells, the surface expression of CD25 on B cells was significantly affected by BLV status (*p* = 0.0044). Only B cells from BLV^−^ cows demonstrated higher CD25 MFI after P/I stimulation; B cells from BLV^+^ cows actually decreased CD25 surface expression after KLH stimulation, and the surface expression of CD25 was lower on B cells from BLV^+^ cows, in comparison to B cells from BLV^−^ cows, after both KLH and P/I stimulation (Figure 3B).

**Figure 4:**
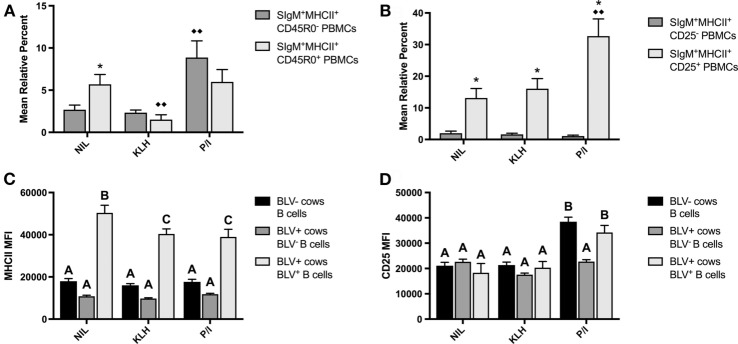
BLV expression *in vitro*. PBMCs from BLV^+^ and BLV^−^ cows were cultured in the presence of no (NIL), keyhole limpet hemocyanin (KLH), or positive control (P/I) stimulation. **(A)** Mean relative percent of BLV^+^SIgM^+^MHCII^+^CD45R0^−^ or BLV^+^SIgM^+^MHCII^+^CD45R0^+^ B cells from BLV^+^ cows. **(B)** Mean relative percent of BLV^+^SIgM^+^MHCII^+^CD25^−^ or BLV^+^SIgM^+^MHCII^+^CD25^+^ B cells from BLV^+^ cows. **(C)** MHCII mean fluorescence intensity (MFI) on SIgM^+^MHCII^+^ B cells from BLV^−^ cows, SIgM^+^MHCII^+^BLV^−^ B cells from BLV^+^ cows and SIgM^+^MHCII^+^BLV^+^ B cells from BLV^+^ cows. **(D)** CD25 MFI on SIgM^+^MHCII^+^ B cells from BLV^−^ cows, SIgM^+^MHCII^+^CD25^+^BLV^−^ B cells from BLV^+^ cows, and SIgM^+^MHCII^+^CD25^+^BLV^+^ B cells from BLV^+^ cows. *p < 0.05 compared to CD45R0^−^ or CD25^−^ B cells, ^♦♦^*p* < 0.05 compared to nil. Different letters denote significant differences. *n* = 8–9/group. Data represent the mean ± SEM.

PBMCs were also cultured to investigate if KLH stimulation could induce BLV expression in B-cell populations from BLV^+^ cows. KLH failed to trigger BLV expression in any measured B-cell subset. Surprisingly, KLH stimulation actually reduced the proportion of BLV-expressing CD45R0^+^ B cells. However, CD45R0 expression on B cells did not have a significant effect on BLV expression overall (*p* = 0.8236) (Figure 4A). In contrast, CD25 expression on B cells did have a significant effect on BLV expression (*p* = 0.0013); CD25^+^ B cells under all culture conditions exhibited a much greater proportion of BLV expression in comparison to CD25^−^ B cells (Figure 4B).

We also explored how BLV expression affected B-cell phenotypes *in vitro*. When comparing B cells from BLV^−^ cows to B cells from BLV^+^ cows that did (BLV^+^) or did not (BLV^−^) express BLV protein gp51, BLV^+^ B cells expressed much higher MHCII in all culture conditions in comparison to both BLV^−^ B cells and B cells from BLV^−^ cows (Figure 4C). When investigating the effect of BLV expression on CD25 surface expression, CD25 MFI increased in P/I-stimulated cultures as expected, but only on B cells from BLV^−^ cows and BLV^+^ B cells from BLV^+^ cows; BLV^−^ B cells from BLV^+^ cows failed to increase the CD25 MFI (Figure 4D). Taken together, these results suggest that B cells from BLV^+^ cows are less reactive to *in vitro* stimulation than B cells from BLV^−^ cows; BLV expression *in vitro* is more prevalent in activated B cells; and B cells from BLV^+^ cows demonstrate different phenotypes in comparison to B cells from BLV^−^ cows, both in BLV^−^ and BLV^+^ B-cell subsets.

### BLV PVL *In Vivo* Increases after KLH Inoculation

**Figure 5:**
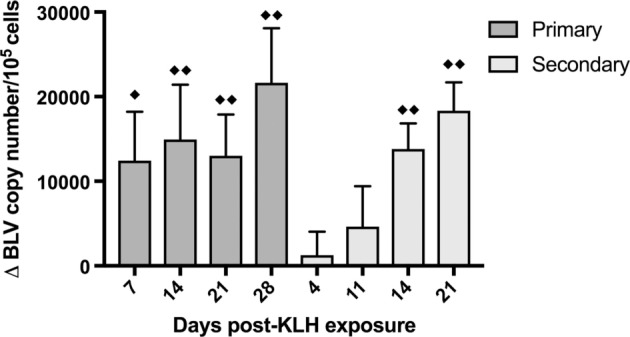
Bovine leukemia virus (BLV) proviral load (PVL) in whole blood in BLV^+^ cows. DNA was extracted from whole blood collected from BLV^+^ cows after keyhole limpet hemocyanin (KLH) inoculation and the BLV PVL was measured. The change in PVL after either primary (d0) or secondary (d56) KLH+ DDA injection was determined. ^♦♦^*p* < 0.05 compared to d0 (for primary) or to d56 (for secondary), ^♦^*p* < 0.1 compared to d0 or d56. *n* = 10. Data represent the mean ± SEM.

In order to assess how the BLV PVL changed over time after KLH+ DDA exposure, DNA was extracted from whole blood from BLV^+^ cattle and qPCR was used to measure PVL. The average BLV PVL on d0 before inoculation was almost 96,000 copies/10^5^ cells; after both primary and secondary KLH inoculations, the PVL sequentially increased over time (*p* < 0.0001) (Figure 5). BLV^+^ cows exhibited the largest increase in PVL on the final day of collection (28 days post-exposure for primary and 21 days post-exposure for secondary); in both cases, the PVL increased by over 18,000 copies/10^5^ cells.

### BLV^+^ Cows Have Reduced Proportions of Circulating T-Cell Populations *In Vivo*

**Figure 6:**
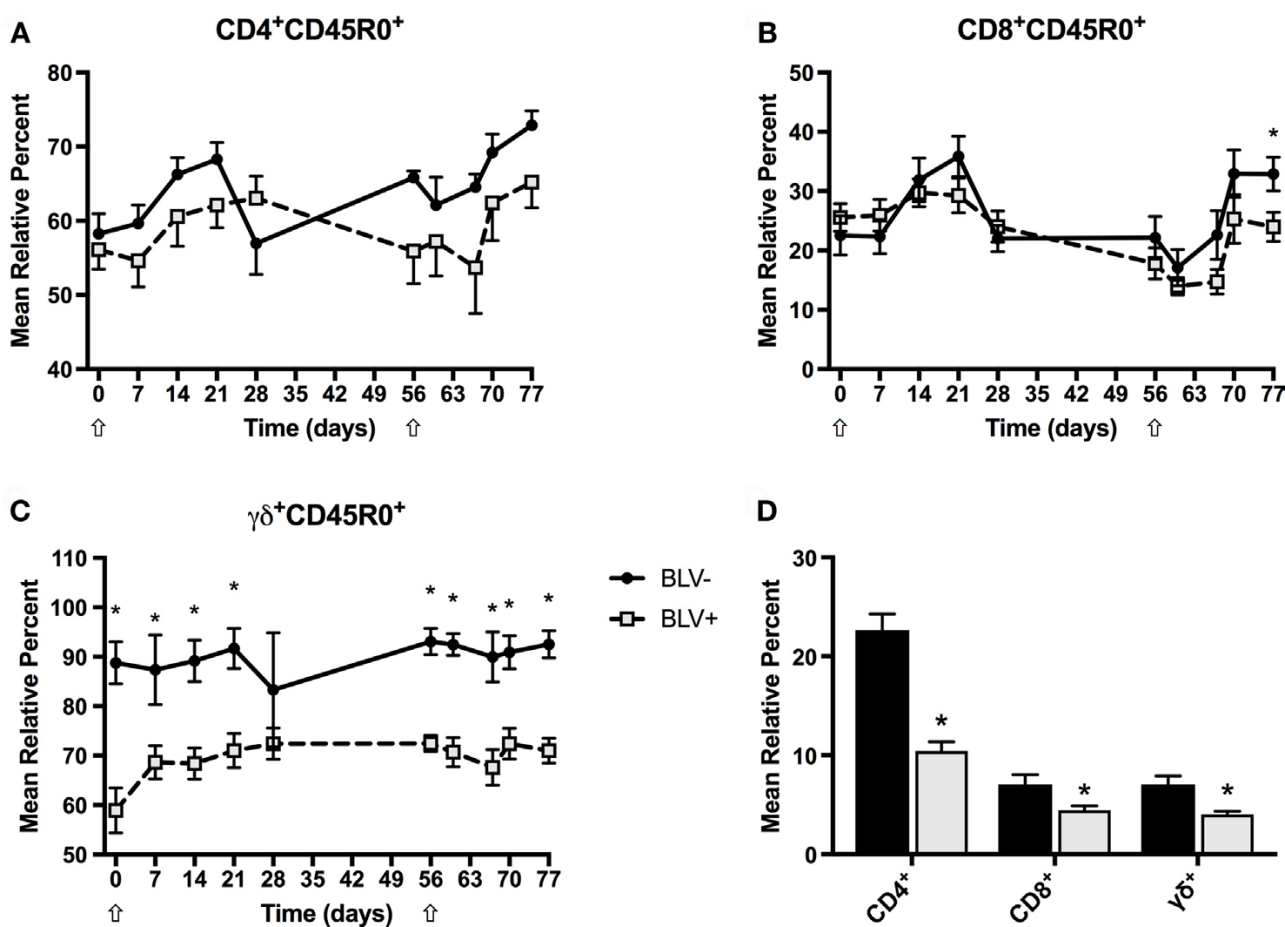
Circulating T-cell populations in BLV^+^ and BLV^−^ cows. Freshly isolated PBMCs from BLV^+^ and BLV^−^ cows were immunostained to characterize the circulating T-cell population. **(A)** Mean relative percent of CD45R0^+^CD4^+^ T cells. **(B)** Mean relative percent of CD45R0^+^CD8^+^ T cells. **(C)** Mean relative percent of CD45R0^+^γδ^+^ T cells. **(D)** Mean relative percent of CD4^+^, CD8^+^, and γδ^+^ T-cell populations on d0. *p < 0.05. *n* = 6–9/group. Arrows denote keyhole limpet hemocyanin inoculations. Data represent the mean ± SEM.

Although BLV most commonly infects B cells, previous research has suggested that T-cell immunity is also compromised in BLV^+^ cows (6). To investigate the circulating effector and memory T-cell population in BLV^+^ cows after KLH inoculation, we labeled freshly isolated PBMCs to measure CD45R0 expression on CD4^+^, CD8^+^, and γδ^+^ T cells. BLV^+^ cows consistently exhibited lower relative proportions of CD4^+^, CD8^+^, and γδ^+^ T cells (*p* < 0.05) (Figure 6D), although these did not change over time. However, both CD4^+^CD45R0^+^ (Figure 6A) and CD8^+^CD45R0^+^ (Figure 6B) T-cell populations significantly responded to primary and secondary KLH inoculations (*p* < 0.05): both populations increased after inoculation, although there was no difference between CD4^+^CD45R0^+^ (*p* = 0.3816) or CD8^+^CD45R0^+^ (*p* = 0.4237) populations in BLV^+^ and BLV^−^ cows. In contrast to classical T cells, the γδ^+^CD45R0^+^ population did not significantly change over time after KLH exposure; however, BLV^+^ cows demonstrated a greatly diminished γδ^+^CD450^+^ T-cell population (*p* < 0.0001) (Figure 6C). These data indicate that while BLV infection does not appear to affect the classical effector/memory T-cell compartment, BLV infection may negatively impact the γδ effector/memory T-cell compartment.

### T Cells from BLV^+^ Cows Produce More IL4 after Stimulation *In Vitro*

**Figure 7:**
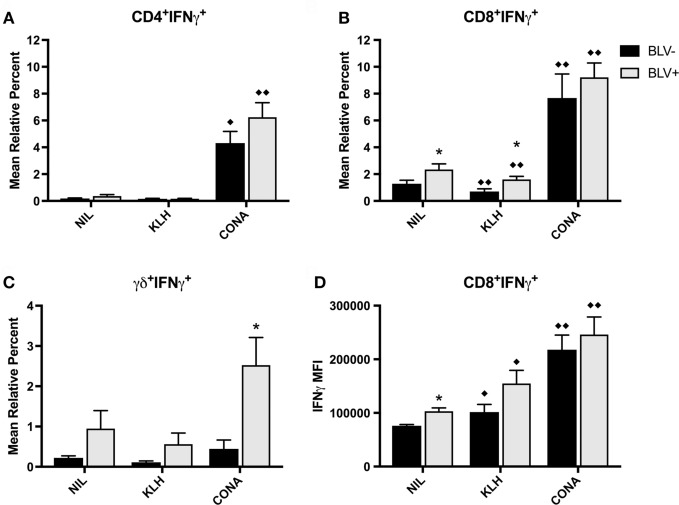
IFNγ production by T cells from BLV^+^ and BLV^−^ cows. PBMCs from BLV^+^ and BLV^−^ cows were cultured with no (NIL), keyhole limpet hemocyanin (KLH), or positive control (CONA) stimulation and IFNγ production by T-cell subsets was measured. **(A)** Mean relative percent of IFNγ^+^CD4^+^ T cells. **(B)** Mean relative percent of IFNγ^+^CD8^+^ T cells. **(C)** Mean relative percent of IFNγ^+^γδ^+^ T cells. **(D)** IFNγ mean fluorescence intensity on CD8^+^IFNγ^+^ T cells. *p < 0.05 compared to BLV^−^, ^♦♦^*p* < 0.05 compared to nil, ^♦^*p* < 0.1 compared to nil. *n* = 8–9/group. Data represent the mean ± SEM.

In addition to investigate the circulating effector/memory T-cell compartment, we examined whether T cells from BLV^+^ cows generated IFNγ or IL4 in response to stimulation *in vitro*. While *in vitro* T-cell activation was measured on d7, 14, 56, 67, and 77, there was no difference between time points; thus, the IFNγ data presented in Figure 7 is from d56. Overall, KLH stimulation failed to increase the proportion of IFNγ^+^ cells in any T-cell subset and actually decreased the proportion of IFNγ^+^CD8^+^ T cells from both BLV^+^ and BLV^−^ cows. There was no overall difference between BLV^+^ and BLV^−^ cows in the proportion of IFNγ^+^CD4^+^ T cells (*p* = 0.150) (Figure 7A) or CD8^+^ T cells (*p* = 0.112) (Figure 7B). Unlike classical T cells, γδ^+^ T-cell IFNγ production was significantly higher in BLV^+^ cows (*p* = 0.0007) (Figure 7C).

We also measured the IFNγ MFI as a measure of reactivity to *in vitro* stimulation. While there was no difference in IFNγ MFI from CD4^+^ or γδ^+^ T cells between BLV^+^ and BLV^−^ cows (data not shown), BLV status did have a significant effect on IFNγ MFI from CD8^+^ T cells (*p* = 0.044): reactive CD8^+^ T cells from BLV^+^ cows were actually producing more IFNγ than reactive CD8^+^ T cells from BLV^−^ cows (Figure 7D).

**Figure 8:**
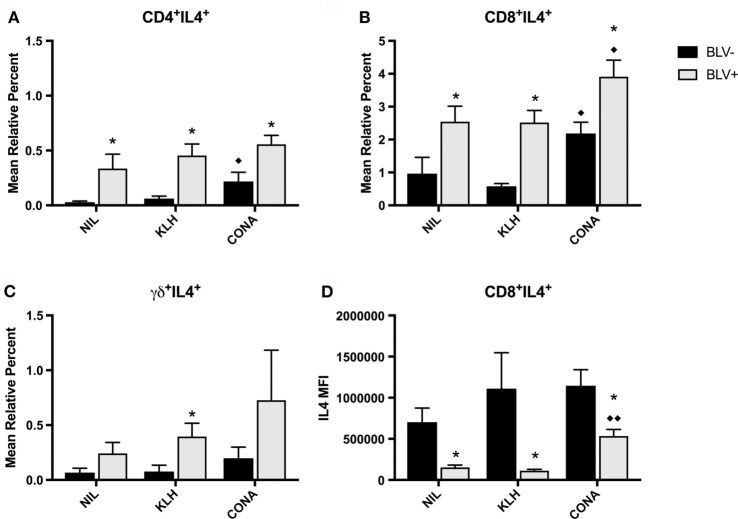
IL4 production by T cells from BLV^+^ and BLV^−^ cows. PBMCs from BLV^+^ and BLV^−^ cows were cultured with no (NIL), keyhole limpet hemocyanin (KLH), or positive control (CONA) stimulation and IL4 production by T-cell subsets was measured. **(A)** Mean relative percent of IL4^+^CD4^+^ T cells. **(B)** Mean relative percent of IL4^+^CD8^+^ T cells. **(C)** Mean relative percent of IL4^+^γδ^+^ T cells. **(D)** IL4 mean fluorescence intensity on CD8^+^IL4^+^ T cells. *p < 0.05 compared to BLV^−^, ^♦♦^*p* < 0.05 compared to nil, ^♦^*p* < 0.1 compared to nil. *n* = 7–8/group. Data represent the mean ± SEM.

We also studied IL4 production by T-cell subsets in BLV^+^ cows. Similar to the *in vitro* IFNγ results, *in vitro* IL4 production was not different between time points; thus, the IL4 data presented in Figure 8 are from d77. Surprisingly, *in vitro* IL4 production was not similar to what was observed with *in vitro* IFNγ production. BLV status had a significant or trending significant effect on the proportion of IL4-producing cells within CD4^+^ (*p* = 0.0009), CD8^+^ (*p* = 0.0006), and γδ^+^ T-cell (*p* = 0.0879) populations. In all three T-cell populations, BLV^+^ cows consistently exhibited a higher proportion of IL4-producing T cells (Figures 8A–C). However, reactive CD8^+^ T cells from BLV^+^ cows actually exhibited lower IL4 expression in comparison to reactive CD8^+^ T cells from BLV^−^ cows (*p* < 0.0001) (Figure 8D). The IL4 MFI from CD4^+^ and γδ^+^ T cells was not different between BLV^+^ and BLV^−^ cows (data not shown). Taken together, these results imply that T cells from BLV^+^ cows are capable of producing both IFNγ and IL4 after *in vitro* cell culture; however, the balance in BLV^+^ cows may favor a greater proportion of IL4-producing T cells.

## Discussion

In this study, we investigated how BLV^+^ dairy cows respond to both a primary immune stimulation and a secondary immune stimulation. After exposing BLV^+^ and BLV^−^ cows to a non-infectious antigen (KLH), both B- and T-cell responses were tracked over a 3-month period to measure both the primary and secondary immune responses. Alterations were observed in both B- and T-cell immunities in BLV^+^ cows relative to uninfected herdmates: BLV^+^ cows produced lower titers of KLH-specific IgM after primary immune stimulation; exhibited fewer CD45R0^+^ B cells, increased CD5^dim+^ B cells with higher CD5 expression, reduced CD5 expression on CD5^bright+^ B cells, and reduced MHCII surface expression on B cells in circulation; displayed reduced B-cell activation *in vitro*; and exhibited an increase in BLV PVL. In addition, BLV^+^ cows demonstrated a reduced CD45R0^+^γδ^+^ T-cell population in the periphery and demonstrated a greater prevalence of IL4-producing T cells *in vitro*. Taken together, these results suggest that BLV^+^ cows do have abnormal immune responses even upon a primary immune stimulation. Atypical immune responses may make BLV^+^ cows more vulnerable to other infections of economic importance. This risk is readily apparent at first exposure to a novel antigen as well as after repeated exposure to a common vaccine (10).

Similar to results seen in previous studies (10, 11), BLV^+^ cows demonstrated less antigen-specific IgM after primary stimulation. Although BLV^+^ cows produced equivalent levels of IgM after secondary stimulation, this appears to be the result of IgM levels declining in BLV^−^ cows between primary and secondary stimulations. In contrast, no differences were observed in either IgG1 production or IgG2 production against KLH. While other studies have found altered IgG levels in BLV^+^ cattle (9, 11), it is possible that any impairment of IgG production is only detectable after repeated antigen exposure. While IgG antibodies have higher affinity for antigens, IgM is particularly important during a primary immune response as the first secreted isotype and is especially important for activating complement, which provides critical protection against bacterial infections.

It was interesting to note that the overall circulating B-cell population in BLV^+^ cows alone increased after both primary and secondary KLH stimulations. 30% of BLV^+^ cattle develop PL, which is characterized by a chronic, polyclonal expansion of B cells in peripheral blood (3). While the mechanisms by which BLV causes PL are not well understood, B-cell population dynamics are clearly dysregulated during polyclonal expansion (6). Because the prevalence of circulating B cells in BLV^+^ cows did not remain elevated between primary and secondary stimulations, it suggests that the prevalence of circulating B cells in the periphery was the result of KLH^+^ DDA exposure. It was especially intriguing that the BLV PVL significantly increased over time after both the primary and secondary KLH inoculations. BLV primarily infects B cells (5) and prior research found that 66% of CD5^+^ B cells carried the provirus (16), so these results could indicate that BLV-infected B cells proliferate in response to immune stimulation.

In addition to the total B-cell population, two subpopulations of circulating B cells were tracked. Although CD45R0^+^ B cells did not change over time, BLV^+^ cows consistently demonstrated a substantial reduction in the prevalence of CD45R0^+^ B cells. While CD45R0 expression is better characterized on αβ T cells, its expression on B cells likely indicates either (1) a memory B cell or (2) a differentiating B cell that may become either a memory B cell or a plasma cell (17). The smaller CD45R0^+^ B-cell population in BLV^+^ cows at all time points could indicate a deficiency in either developing or maintaining B-cell memory, which would be especially detrimental for any immune response dependent on antibody production. Another concerning result was that the overall B-cell population, and the CD5^dim+^ B-cell population in particular, in BLV^+^ cows had lower MHCII surface expression, which could also impair humoral immunity development that is required for isotype switching. Although in this study we did not observe reduced IgG1 or IgG2 antibody production in BLV^+^ cows, this may be because we only tested a secondary antigen exposure. A previous study has found a reduced IgG2 antibody production in BLV^+^ cows after repeated vaccination (9).

When examining the CD5^+^ B-cell compartment, we detected two distinct CD5^+^ B-cell populations, which we denoted CD5^dim+^ and CD5^bright+^. While bovine B cells have been found to contain both a CD5^dim^ population and a CD5^bright^ population (18), most BLV studies have only focused on CD5 expression in total, where CD5^+^ B cells are the type of B cell that expands in PL cattle (5). However, we kept the CD5^dim+^ and CD5^bright+^ B-cell populations distinct because the two populations showed different kinetics after KLH inoculation and there were significant differences in these two cell populations between BLV^+^ and BLV^−^ cows. While the CD5^dim+^ B-cell population increased after KLH inoculation, the CD5^bright+^ B-cell population sharply declined. When comparing BLV^+^ and BLV^−^ cows, BLV^+^ cows had an elevated CD5^dim+^ population, but the CD5^bright+^ populations were equivalent. Finally, while CD5^dim+^ B cells from BLV^+^ cows exhibited higher mean CD5 surface expression, CD5^bright+^ B cells from BLV^+^ cows exhibited lower mean CD5 surface expression. It is unclear what functional differences may exist between CD5^dim+^ and CD5^bright+^ B cells. CD5 expression on B cells may be a lineage marker for innate-like B cells that produce natural IgM (19), but CD5 expression on B cells may also be induced after stimulation through the B-cell receptor (20), and it is possible that the CD5^dim+^ and CD5^bright+^ populations reflect these different patterns of CD5 expression. Our data suggests that the distinction is relevant in the context of BLV infection, but our study was unable elucidate the function of these distinct B-cell subtypes.

We also investigated how B cells responded to KLH stimulation *in vitro*. While B cells from both BLV^+^ and BLV^−^ cows showed a minor but significant increase in CD25^+^ B cells after KLH stimulation, the proportion of CD25^+^ B cells from BLV^+^ cows was significantly lower. In addition to the relative percent of CD25^+^ B cells, CD25^+^ B cells from BLV^+^ cows exhibited significantly lower CD25 surface expression after both KLH and P/I stimulations, suggesting that BLV^+^ cows display less antigen-specific or mitogenic B-cell activation when compared to healthy, BLV^−^ cows. We also questioned whether KLH stimulation would induce BLV protein expression. While P/I stimulation induced BLV gp51 expression as expected (21), KLH stimulation did not. However, it was clear that BLV expression was most common in CD25^+^ B cells, indicating that BLV gp51 expression is related to B-cell activation. BLV expression was also related to different patterns of MHCII and CD25 surface expression. Under all culture conditions, BLV-expressing B cells had a dramatically increased MHCII surface expression, which was in contrast to MHCII expression observed on B cells *ex vivo*. Active BLV transcription is rarely detected *ex vivo*, while even short term *in vitro* culture can induce detectable BLV protein production (22), which suggests that the BLV protein expression is inducing elevated MHCII expression. In contrast, BLV expression did not affect CD25 surface expression in unstimulated or KLH-stimulated cultures. After P/I stimulation, CD25 surface expression increased only on B cells from BLV^−^ cows or on BLV-expressing B cells from BLV^+^ cows; the CD25 surface expression on BLV^−^ B cells from BLV^+^ cows remained unchanged. Overall, our B-cell culture experiments suggest that B cells from BLV^+^ cows have a reduced responsiveness to antigenic and mitogenic stimulations and this reduced responsiveness is not necessarily dependent on active BLV protein expression.

While the potential effect of BLV infection on B cells is obvious, it is less clear how BLV infection affects the T-cell compartment, although T-cell irregularities have been previously observed (6). We measured the circulating CD45R0^+^ T-cell populations to investigate effector/memory T cells (23). Both the CD4^+^ and CD8^+^ CD45R0^+^ T-cell populations increased over time after KLH+ DDA stimulation, although the abundance of the cell types was equivalent between BLV^+^ and BLV^−^ cows. While the γδ^+^CD45R0^+^ T-cell population remained mostly constant after KLH+ DDA exposure, BLV^+^ cows exhibited a large overall reduction in their γδ^+^CD45R0^+^ T-cell population. Although γδ^+^CD45R0^+^ T cells are not a well-characterized cell population, CD45R0 expression likely indicates current or prior activation, although it is unclear whether this activation would be innate or adaptive or both (24).

We were also interested in investigating IFNγ and IL4 productions in response to KLH stimulation *in vitro*. While we were unable to detect increased cytokine production in KLH-stimulated cell culture, we found that both CD4^+^ and CD8^+^ T-cell populations from BLV^+^ and BLV^−^ cows were equally responsive to positive control stimulation when measuring IFNγ production. Similar to previous results (10), a significantly higher proportion of γδ^+^ T cells from BLV^+^ cows produced IFNγ in cell culture. Surprisingly, while the amount of IFNγ produced by CD4^+^ and γδ^+^ T cells was not different between BLV^+^ and BLV^−^ cows, CD8^+^ T cells from BLV^+^ cows did produce significantly higher amounts of IFNγ. When we similarly investigated IL4 production *in vitro*, we observed directly opposite results. CD4^+^ and CD8^+^ T-cell populations from BLV^+^ cows contained higher proportions of IL4-producting cells under all cell culture conditions; conversely, γδ^+^ T cells produced IL4 in equivalent proportions between BLV^+^ and BLV^−^ cows. Finally, while BLV^+^ cows contained higher proportions of IL4-producing CD8^+^ T cells *in vitro*, reactive CD8^+^ IL4-producing cells from BLV^−^ cows actually produced more IL4 under all culture conditions. This difference in IL4-producing cells versus IL4 expression on a per-cell basis could be a result of suppressed activation in CD8^+^ T cells from BLV^+^ cows. While BLV^+^ cows have a higher proportion of CD8^+^ T cells producing IL4, it is possible that BLV infection interferes with the degree of activation after CD8^+^ T cell stimulation, although this was not observed with IFNγ production in CD8^+^ T cells from BLV^+^ cows. While IL4 production by CD8^+^ T cells in cattle is not well-studied, evidence from humans and mice suggest that CD8^+^ T cells may also be polarized based upon their cytokine secretion and that this cytokine secretion can contribute to the overall balance of a cell-mediated versus humoral immune response (25, 26).

While our experiment was unable to detect any differences in antigen-specific activation in T cells from BLV^+^ cows, we did detect overall differences in circulating γδ^+^ T cells and in αβ and γδ^+^ T-cell cytokine production *in vitro*. The considerable reduction in circulating CD45R0^+^γδ^+^ T cells in BLV^+^ cows could suggest an impairment of effector or memory γδ^+^ T cells in BLV^+^ cows; considering the hypothesized importance of γδ^+^ T cells in bovine immunity (27), an impairment in this T-cell subtype could have serious consequences on both innate and adaptive immunities in cattle, including reduced responsiveness to vaccination and less immune protection from pathogens including *Mycobacterium bovis* and *Leptospira borgpetersenii* serovar Hardjo (28). Our *in vitro* experiments suggest that αβ T cells in BLV^+^ cattle could be more predisposed to produce IL4 as compared to αβ T cells from healthy, BLV^−^ cattle. If BLV^+^ cattle immunity is more skewed toward IL4 over IFNγ production, this could have profound effects upon infections that depend on Th1 versus Th2 immunity for effective pathogen clearance.

This study was conducted to investigate B- and T-cell responses in BLV^+^ cows to a primary and secondary antigenic immune challenge. While evidence demonstrates that BLV-infected cattle have atypical immunity in comparison to BLV^−^ cows (6), little research has investigated whether BLV^+^ cows would exhibit abnormal immune responses to a primary challenge, or if abnormal adaptive immunity in BLV^+^ cattle was the cumulative effect of multiple antigenic challenges. Our study did find antigen-specific deficiencies in B-cell immunity during a primary immune response, indicating that BLV infection can interfere with antigen-specific immunity without many re-exposures to antigen. While we were unable to detect antigen-specific T-cell responses *in vitro*, we did detect abnormalities in circulating γδ^+^ T cells in BLV^+^ cows, as well as a potential bias for IL4-producing αβ T cells. These data demonstrate that BLV infection can have a detectable impact on immune stimulation even upon a primary antigen exposure, which would likely mean that negative impacts of BLV infection on herd health could occur immediately upon a secondary infection.

## Ethics Statement

All protocols were reviewed and approved by the Michigan State University Institutional Animal Use and Care Committee (AUF# 04/15-061-00).

## Author Contributions

MF led the study and was involved in the study conception, design, execution, analysis, and interpretation of data. MF wrote the manuscript. KS contributed to study design, execution and interpretation of data, and manuscript editing. OB contributed to the study execution and analysis of data and wrote the PVL methods section of the manuscript. JW contributed to the study execution and manuscript editing. CD contributed to the study execution and manuscript editing. PB contributed to the study conception and design and manuscript editing. PC contributed to the study conception, design, and interpretation and manuscript editing.

## Conflict of Interest Statement

The authors declare no conflict of interest. NorthStar Cooperative is a for-profit animal agriculture diagnostic company and conducted all diagnostic testing for this study free of charge in exchange for biological samples.

## Supplementary Material

Figure S1 Gating strategy to determine CD5^dim+^ and CD5^bright+^ B cells. To identify CD5^+^ dim and bright populations, the upper right quadrant was used to denote CD5^+^ cells. A rectangle gate was used to select CD5^bright+^ cells, and cells inside the quadrant gate and outside the rectangle gate were determined to be CD5^dim+^ cells. Representative plots are from fresh labeling on D56 and feature samples from representative BLV^−^ and BLV^+^ cows.
