# Supplementary figures and images for: Dairy Cows Naturally Infected with Bovine Leukemia Virus Exhibit Abnormal B- and T-Cell Phenotypes after Primary and Secondary Exposures to Keyhole Limpet Hemocyanin
Source: Front Vet Sci. 2017 Jul 14;4:112. doi: 10.3389/fvets.2017.00112 (PMC5509956; doi:10.3389/fvets.2017.00112)

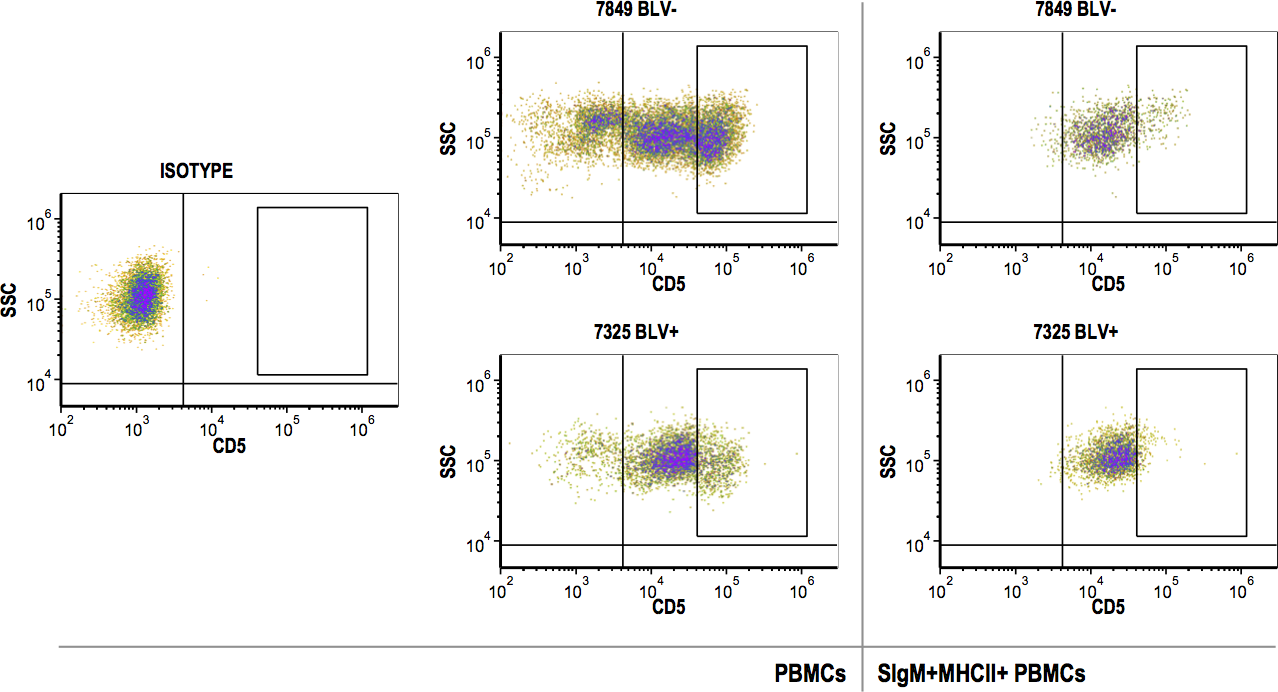

Supplement: Figure S1 — Gating strategy to determine CD5dim+ and CD5bright+ B cells. To identify CD5+ dim and bright populations, the upper right quadrant was used to denote CD5+ cells. A rectangle gate was used to select CD5bright+ cells, and cells inside the quadrant gate and outside the rectangle gate were determined to be CD5dim+ cells. Representative plots are from fresh labeling on D56 and feature samples from representative BLV− and BLV+ cows. [file Image_1.TIFF]
